# A computer-aided determining method for the myometrial infiltration depth of early endometrial cancer on MRI images

**DOI:** 10.1186/s12938-023-01169-w

**Published:** 2023-10-31

**Authors:** Liu Xiong, Chunxia Chen, Yongping Lin, Wei Mao, Zhiyu Song

**Affiliations:** 1 School of Optoelectronic and Communication Engineering, Xiamen University of Technology, No. 600 Ligong Road, Jimei District, Xiamen, 361024 Fujian China; 2 Fujian Maternity and Child Health Hospital, College of Clinical Medicine for Obstetrics & Gynecology and Pediatrics, Fujian Medical University, No.18 Daoshan Road, Gulou District, Fuzhou, 350001 Fujian China

**Keywords:** Computer-aided diagnosis, Deep learning, Magnetic resonance imaging, Uterine cavity line

## Abstract

To classify early endometrial cancer (EC) on sagittal T2-weighted images (T2WI) by determining the depth of myometrial infiltration (MI) using a computer-aided diagnosis (CAD) method based on a multi-stage deep learning (DL) model. This study retrospectively investigated 154 patients with pathologically proven early EC at the institution between January 1, 2018, and December 31, 2020. Of these patients, 75 were in the International Federation of Gynecology and Obstetrics (FIGO) stage IA and 79 were in FIGO stage IB. An SSD-based detection model and an Attention U-net-based segmentation model were trained to select, crop, and segment magnetic resonance imaging (MRl) images. Then, an ellipse fitting algorithm was used to generate a uterine cavity line (UCL) to obtain MI depth for classification. In the independent test datasets, the uterus and tumor detection model achieves an average precision rate of 98.70% and 87.93%, respectively. Selecting the optimal MRI slices method yields an accuracy of 97.83%. The uterus and tumor segmentation model with mean IOU of 0.738 and 0.655, mean PA of 0.867 and 0.749, and mean DSC of 0.845 and 0.779, respectively. Finally, the CAD method based on the calculated MI depth reaches an accuracy of 86.9%, a sensitivity of 81.8%, and a specificity of 91.7% for early EC classification. In this study, the CAD method implements an end-to-end early EC classification and is found to be on par with radiologists in terms of performance. It is more intuitive and interpretable than previous DL-based CAD methods.

## Introduction

Endometrial carcinoma, or uterine cancer, is a malignancy arising from the endometrium [1]. Women have a 1 in 40 lifetime risk of being diagnosed with endometrial cancer (EC), which is regarded as the fourth most common malignancy among women. Furthermore, in countries with rapid socioeconomic transition, its incidence rate has increased over time and in successive generations [2]. According to the data of Global Cancer Statistics in 2018 and 2020, the number of new cases of corpus uteri cancer was 382,069 and 417,367, respectively, and the number of deaths was 89,929 and 97,370, respectively [3, 4]. American Cancer Society estimates the number of new cases of uterine corpus cancer will be 66570, and the deaths will be 12940 in the US, in 2021. The uterine corpus cancer is often referred to as EC because more than 90% of cases occur in the endometrium (lining of the uterus) [5]. According to the International Federation of Gynecology and Obstetric (FIGO) in 2009, carcinoma of the endometrium is divided into 4 stages [6]. Most ECs (75%) are diagnosed at an early stage (FIGO stages I or II) and the 5-year overall survival ranges from 74% to 91%, but for FIGO stage III and IV, the 5-year overall survival is only 57% to 66% and 20% to 26% [7]. Although tumors can be graded by preoperative endometrial biopsy, this method may underestimate the grade of the tumor compared to the final surgical pathology [8, 9]. Prognostic factors such as FIGO staging, histological grade, and lymph node metastasis (LNM), which are used for risk stratification, can usually only be assessed in the surgical specimen [8–10]. Computer-aided diagnosis (CAD) in medical imaging aims to assist specialists in diagnosing diseases [11]. Therefore, a non-invasive method for predicting the staging and invasiveness of tumors that can help radiologists to risk-stratify early EC patients is a clinical need.

Preoperative imaging is essential for the surgical management of EC, and pelvic magnetic resonance imaging (MRI) is preferred for assessing the extent of localized tumors in the pelvis [12]. MRI can accurately outline the extent of localized disease and depict the spread of tumors outside the uterus, and is highly sensitive and specific for describing important prognostic factors [13, 14]. In recent years, there has been increasingly attention to employing CAD methods based on machine learning (ML) to help radiologists analyze MRI images of EC patients. Examples include assessment of the depth of myometrial infiltration (MI) [15, 16], classification of stage IA and stage IB in patients with FIGO stage I [17], and detection of LNM [18, 19]. In Chen et al.’s study, a YOLOv3 model was used to detect uterine and tumor regions, and then the detected regions were cropped out and fed into a CNN model for classification, with an Accuracy (ACC) of 84.78% with the Sensitivity (SEN) of 66.67% and the Specificity (SPE) of 87.50% [15]. Dong et al. used a U-net model with different encoder structures to semantically segment the uterine and tumor regions on MRI, then manually annotated the uterine cavity lines of the segmented maps, which in turn assessed the depth of MI of the EC patient, with a classification ACC of 79.2% on T1-weighted imaging (T1WI) and 70.8% on T2-weighted imaging (T2WI) [20]. For the detection of lymph nodes, Bnouni et al. used a region-growing algorithm to segment the region of interest (ROI) and then used a support vector machine (SVM) to classify the segmented ROI regions with an ACC of 78.50% [18]. Similarly, Yang et al. also chose the decision-tree classification method and achieved SEN and SPE and area under curve (AUC) of 86%, 78%, and 0.85, respectively [19].

Research to date has reported relatively unsatisfactory SEN, SPE and ACC for MI assessment on MRI images using CAD methods based on the ML approach. A sequence of MRI images is generated after a patient undergoes an MR examination. However, typically only one or two slices of these images can reflect the lesion. Previous studies usually required radiologists to manually select that slice, which is a time-consuming and error-prone task. The results of MI assessment depend mainly on the extraction of MRI image features and the lack of the radiologist’s view. In order to address these limitations, this study proposed a method to generate a virtual uterine cavity line (UCL) (i.e., the presumed inner edge of the myometrium) to assess the depth of MI of the tumor. The main idea focuses on determining the depth of MI in FIGO stage I EC. To our best knowledge, no study has been published on combining DL and UCL on MRI images to automatically determine MI depth.

Therefore, the aims of this study are as follows:

1. Establish an object detection network to automatically ROI and select the optimal slice from the patient’s MRI sequence.
2. Establish a semantic segmentation network to segment the uterine and tumor regions on the slices.
3. Employ a uterine cavity line generation algorithm (UCLGA) to generate UCL on the segmentation map. Calculate the exact MI depth and classify early endometrial cancer based on the UCL.

## Results

### Performance of the automatic ROI detection model on MRI

**Table 1:** The detection results of different object detection models

	Average precision (threshold = 0.75)				
	SSD (%)	Fast R-CNN (%)	CenterNet (%)	YOLOv8 (%)	DETR (%)
Uterus	98.70	63.07	60.52	89.56	83.30
Tumors	84.93	1.47	2.49	67.11	56.20

**Fig. 1:**
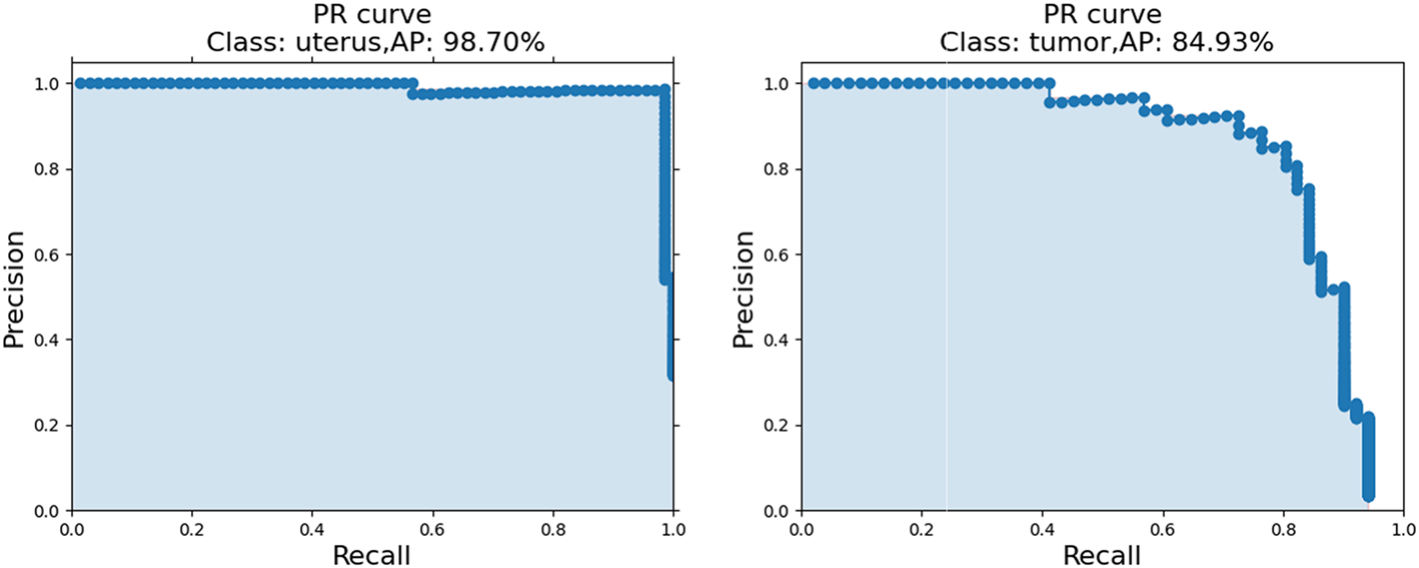
The PR analysis for detecting ROI region. The PR curves for the uterine region (left) and the tumor region (right) in the testing dataset show the corresponding average precision rate of 98.70% and 84.93%

**Fig. 2:**
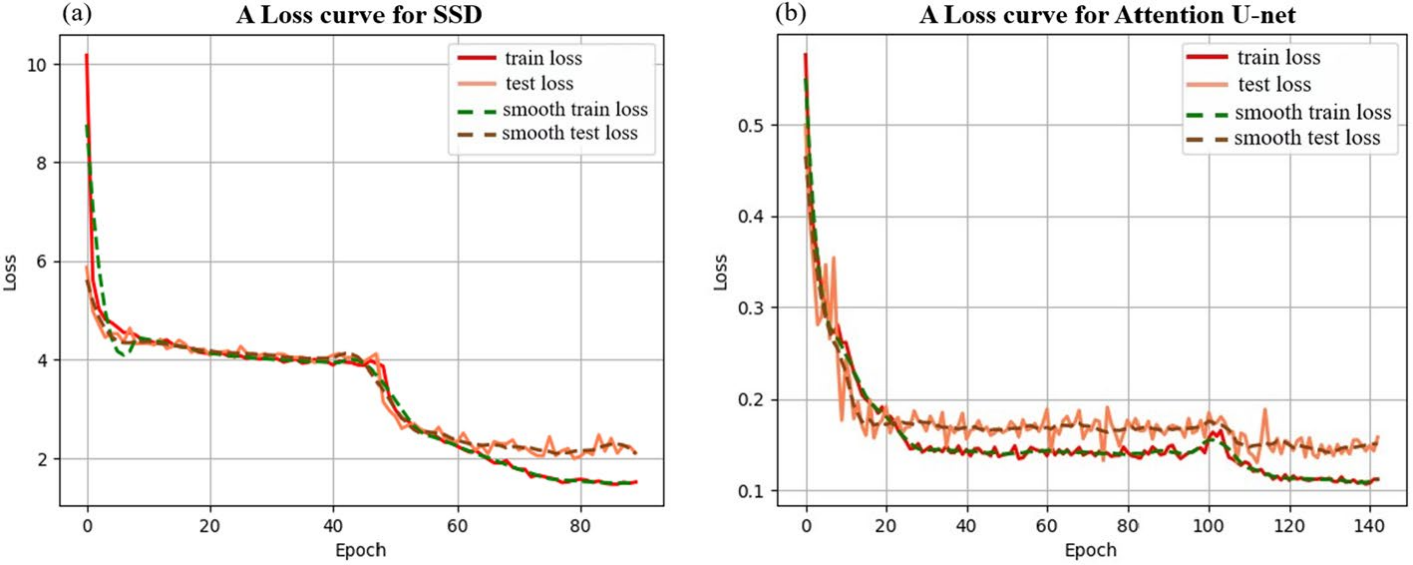
Loss curves for the training and validation sets are provided for each model. **a** A loss curve for SSD model. **b** A loss curve for Attention U-net model

In this study, four other classical object detection models were studied to detect uterine and tumor regions in MRI images. A threshold value of a minimum overlap ratio of 0.75 is used to determine positive samples. The detection results of different object detection models are shown in Table 1. The data in the table show that the SSD model is the best in detecting the uterus and tumor regions, while the other models perform better in detecting only the uterus region. This is due to the fact that the SSD model has the least amount of parameters compared to the other models, which avoids overfitting. In the independent test dataset, the SSD model obtains an average precision (AP) of 98.70 and 84.93% in the uterine region and the tumor region, respectively. The detection results for the uterine region and the tumor region are shown in Fig. 8. The corresponding precision-recall (PR) curves are shown in Fig. 1. The loss curve of the best object detection model is shown in Fig. 2a. The training was performed for 100 epochs with a batch size of 8 and an early stop mechanism. By using the radiologist’s manual selected slices as positive labels, the CAD has a 67.39%, 86.96% and 97.83% accuracy rate in selecting the optimal slice in CAD1-accuracy, CAD2-accuracy and CAD3-accuracy.

### Performance of the semantic segmentation model

The segmentation results for the uterine region and the tumor region are shown in Fig. 9e. The segmentation performance of the model is evaluated using Intersection over Union (IOU), Pixel Accuracy (PA), and Dice Similarity Coefficient (DSC). Two other state-of-the-art semantic segmentation models were used to segment uterine and tumor regions in MRI images. The segmentation results of different models are shown in Table 2, all models have better segmentation results for the uterus than the tumor. This is due to the fact that early lesions are small, and the tumor boundary is not clear enough. Compared to other models, the Attention U-net model has the best segmentation performance. This is because the model is primarily designed for medical datasets and can deliver good results even on small datasets. The loss curve of the best semantic segmentation model is shown in Fig. 2b. The training was performed for 200 epochs with a batch size of 8 and an early stop mechanism.

**Table 2:** The segmentation results of different semantic segmentation models

Model	Region	IOU	PA	DSC
Attention U-net	Tumor (mean ± std)	0.655 ± 0.159	0.749 ± 0.171	0.779 ± 0.127
	Uterus (mean ± std)	0.738 ± 0.099	0.867 ± 0.079	0.845 ± 0.067
SegFormer	Tumor (mean ± std)	0.610 ± 0.293	0.686 ± 0.318	0.705 ± 0.298
	Uterus (mean ± std)	0.715 ± 0.168	0.806 ± 0.173	0.820 ± 0.193
Deeplabv3	Tumor (mean ± std)	0.591 ± 0.260	0.667 ± 0.293	0.701 ± 0.263
	Uterus (mean ± std)	0.722 ± 0.132	0.867 ± 0.112	0.831 ± 0.102

**Fig. 3:**
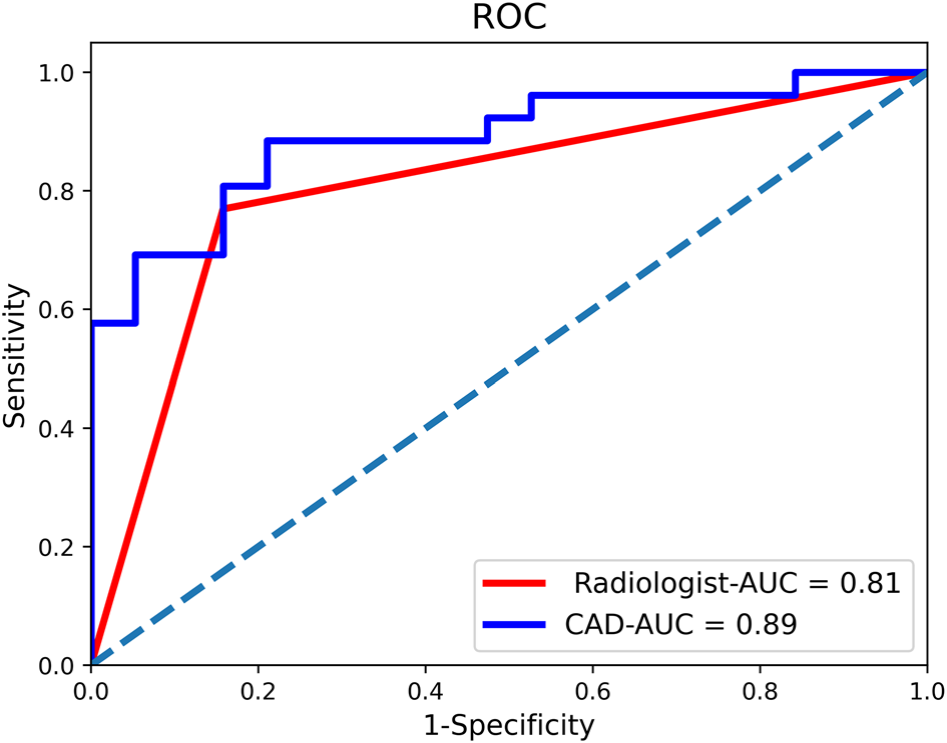
ROC analysis with the CAD for classifying MI depth. The ROC curves for the radiologist (red) and the proposed CAD method (blue) show the AUCs of 0.81 and 0.89, respectively

### Comparison of diagnostic results between CAD and radiologists

**Fig. 4:**
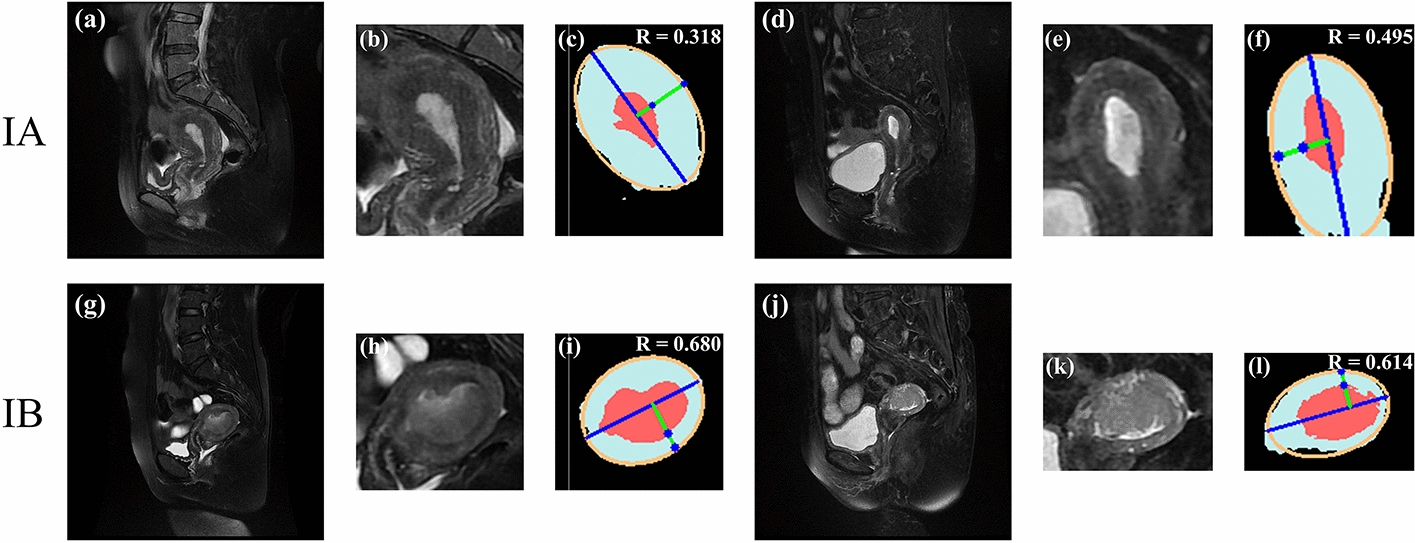
Results of the CAD determination of MI depth on stage IA and IB

Considering stage IA as the positive sample, stage IB as the negative sample, and pathological diagnostic results as the gold standard. By using the receiver operating characteristic curve (ROC) (Fig. 3), the AUC of the proposed CAD method on the test dataset is 0.89, and the AUC of the radiologist on the test dataset is 0.81. The difference in the number of points on the ROC curve is due to the CAD model’s output of classification probabilities, which allows for varying classification outcomes at different probability thresholds, resulting in a greater number of data points. The red line (Radiologist) presents fewer points due to the radiologist providing direct binary classification results, which do not vary based on different probability thresholds. In order to determine the difference between these two AUC values, the study was statistically analyzed by using the DeLong test. The results show a significant difference (p-value less than 0.05) by different methods. The CAD method correctly classifies a greater number of staged cases compared to the radiologist’s diagnosis (Table 3), which obtains ACC, SEN, and SPE of 86.9%, 81.8%, and 91.7%, respectively (Fig. 4). Figure 4 demonstrates the results of CAD in determining MI depth on sagittal T2WI images of four patients, two in stage IA and two in stage IB.

**Table 3:** Diagnostic performance comparison between radiologist and CAD

Results		Pathology report		ACC (%)	SEN (%)	SPE (%)
		IA	IB			
CAD	IA	18	2	86.70	81.00	91.70
	IB	4	22			
Radiologist	IA	19	6	75.60	66.70	85.70
	IB	3	18			

## Discussion

The proposed CAD approach implements an end-to-end diagnostic flow with 81.8% for SEN, 91.7% for SPE, and 86.9% for ACC in the final determination of MI depth. The results indicate that the CAD method and radiologists have been neck and neck for determining MI depth. In addition, it is compared to other ML-based computer models and is more intuitive and interpretable.

In past studies on EC, MRI data used for experiments were commonly selected manually by radiologists, which was a time-consuming and laborious task. In contrast, the proposed method could automatically select the most suitable MRI slices from an MRI image sequence by computer, completing the end-to-end design of the CAD method used for early EC. X. Chen et al. proposed a two-stage DL method based on a CNN for the evaluation of MI depth that yielded a SEN of 66.6%, a SPE of 87.5%, and an ACC of 84.8% [15]. Although they also used an object detection model (based on YOLOv3) for uterine and lesion region detection in MRI images and obtained 86.67% AP, a better detection performance (98.70% AP) was obtained in our proposed model by using the SSD-based detection. Moreover, they directly fed the cropped images into the classifier for MI depth classification, the CAD system cropped the MRI images of detection boxes first, and further performed semantic segmentation on the cropped images and did an accurate MI depth calculation. In the study of Dong et al., a similar approach to generate UCL was applied but the classification ACC reached only up to 79.2% [20]. We speculate that lower ACC might be due to a semantic segmentation model they employed to directly complete the segmentation of the endometrial lining (which we call UCL) on a whole MRI image. Moreover, it is a great challenge for the radiologist to label the dataset as well as for the predictive performance of the model since the UCL is difficult to find on most MRI images. Zhu et al. established an ML model for identifying deep MI, obtaining ACC, SEN, SPE, and F1 scores of 93.7%, 94.7%, 93.3%, and 87.8% [17]. However, the feature extraction used to train the model is tedious (geometric features, first-order histogram-based features, GLCM-based features) and requires human intervention, which is not fully automated. In contrast, the proposed approach can determine the MI depth fully automated.

**Fig. 5:**
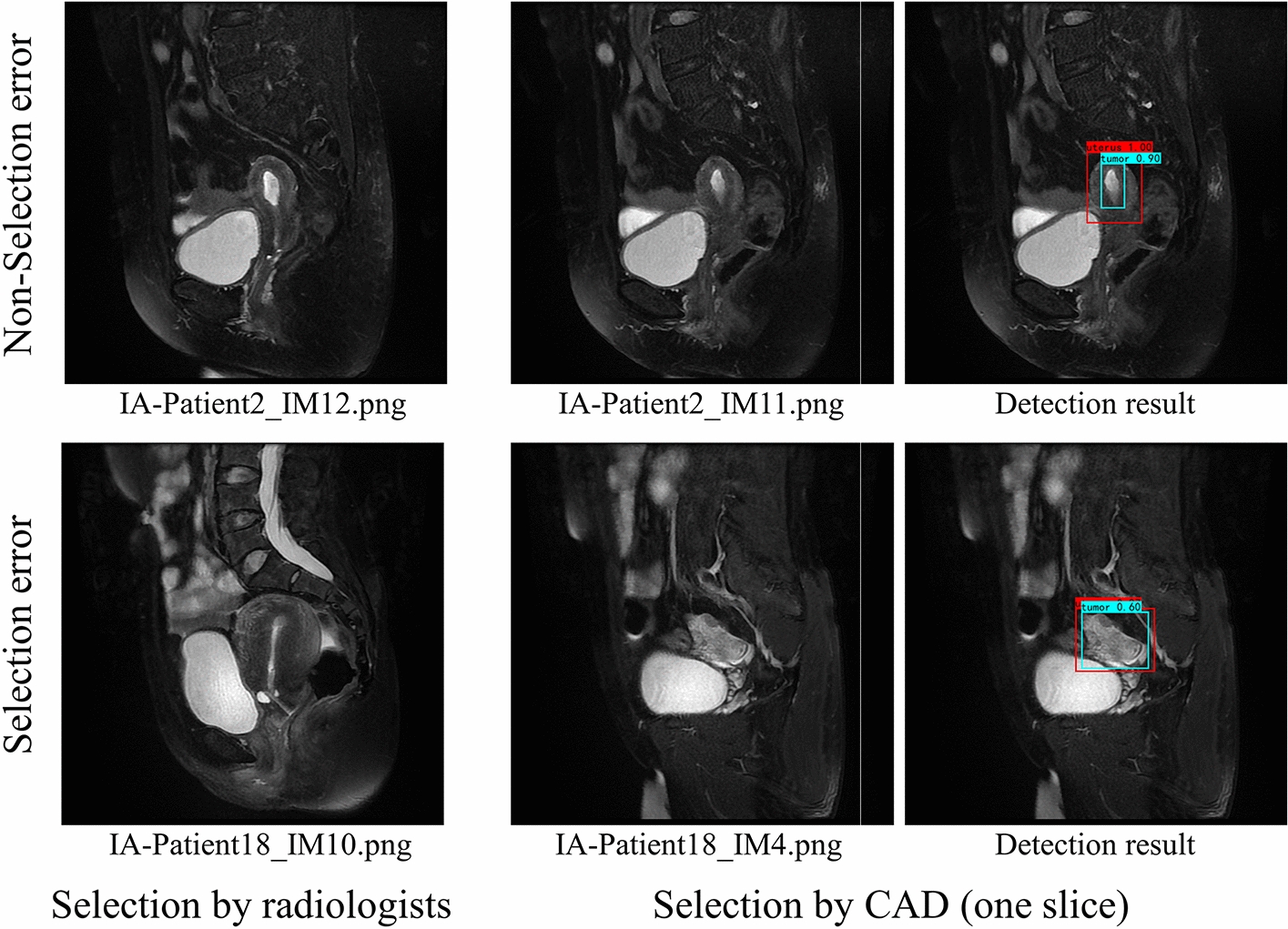
A non-selection error example and a selection error example

The CAD3-accuracy was 97.83% in selecting the optimal MRI slice. Although the CAD2-accuracy and CAD1-accuracy were low, the reason was not about CAD selection errors, but simply non-intersection with the images selected by the radiologists. To obtain the CAD1-accuracy of the IA-Patient2 in the test set as shown in Fig. 5, CAD selected the 11th slice while the radiologist selected the 12th slice. However, the radiologist could also select the 11th slice which is almost not different from the 12th slice. Both slices showed the uterus and tumor correctly. It is not a selection error, just another option by the radiologist which was abandoned randomly, which increases an additional error rate. For the IA-Patient18, the CAD chose the 4th slice and the uterus and tumor were not clearly visible, which was a true selection error. With the exclusion of non-selection errors, CAD1-accuracy increased from 67.39% to 90.48%.

**Fig. 6:**
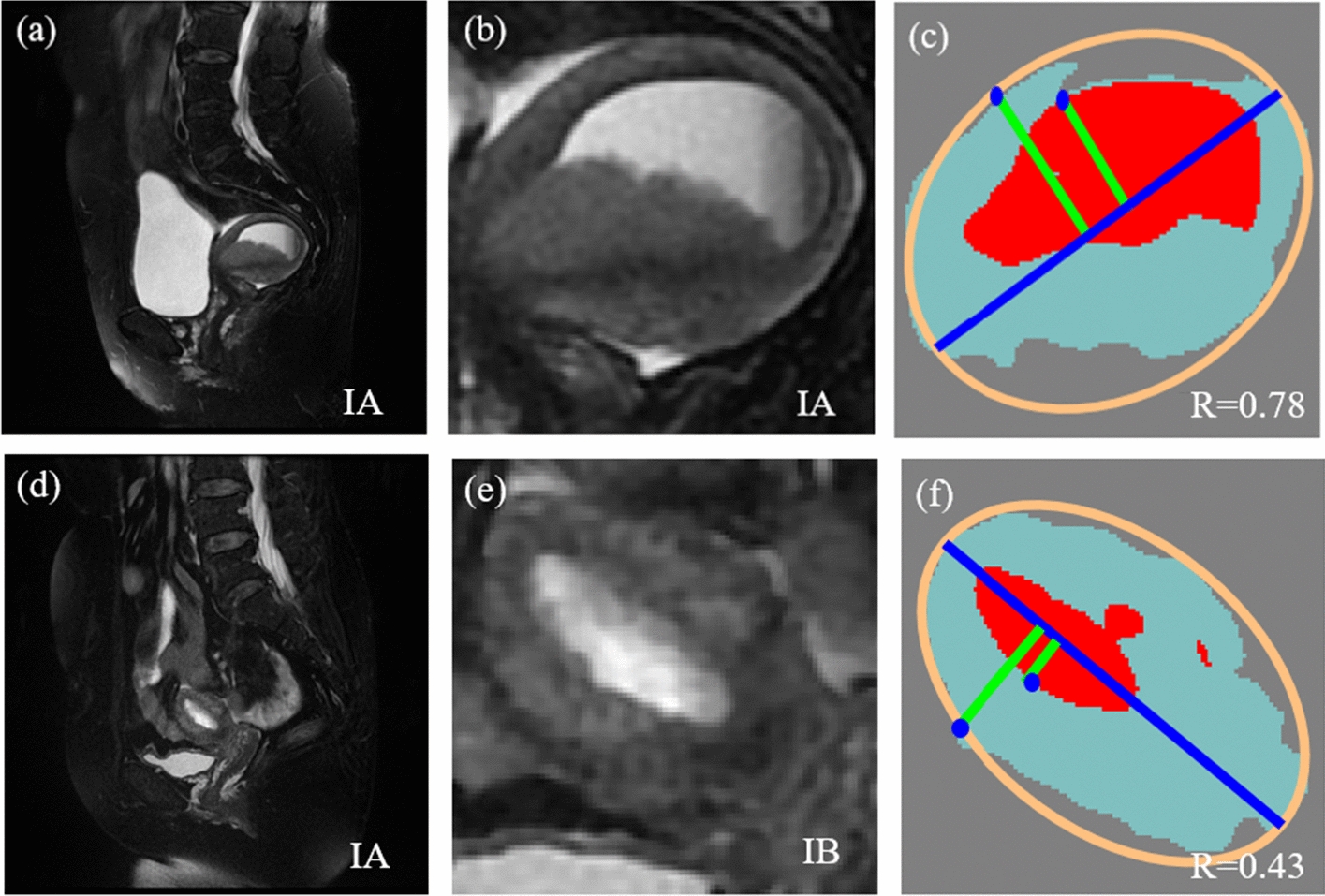
Two examples of diagnostic errors in the proposed method. **a** and **d** Are pathologic staging results. **b** and **e** Are physician diagnoses. **c** and **f** are CAD diagnoses

As shown in Table  3, there is no significant difference in diagnostic performance between CAD methods and radiologists. But there are still mistakes in the final staging as shown in Fig. 6. In the first example (Fig. 6a–c), the radiologist was able to correctly diagnose the case as IA, whereas the CAD method diagnosed it as IB. The lack of precision in tumor and uterine segmentation as well as the fact that the UCL does not correspond to reality leads to diagnostic errors. In the second example (Fig. 6d–f), the radiologist incorrectly diagnosed it as IB while the CAD was able to correctly diagnose it as IA. Although the segmentation is also not very precise, it does not affect the final UCL obtained. Thus, it can be seen that the accurate generation of UCL can greatly improve the accuracy of the diagnosis. For elliptical-shaped and banana-like shaped uterus, the CAD method can better fit the UCL in practice, while for other shapes of the uterus, perhaps a new algorithm is needed to better realize the UCL generation.

It is notable that the cropped MRI images were used in the training and prediction of the semantic segmentation model for two main reasons. One reason is to take full advantage of the object detection model, not only for selecting MRI slices, but also for reducing interference factors that may be inside or outside the uterus (such as pelvic effusion, hematocele, uterine fibroids, cervical cancer, and so on) by using the detection box. The other reason is that the cropped images can reduce the time cost of subsequent model training and prediction, improving the overall efficiency of CAD. An advantage of this study is combining the UCLGA (inspired by the experience of radiologists) with the DL model, which allows us to calculate the exact value of MI depth, visualize the result of our judgments, and interpret it scientifically. Most DL-based studies in the past relied too much on model judgments and could not visualize and explain their classification reasonably. At present, there are few MRI images of patients with advanced EC in the institution, so the advanced-stage EC classification has not been studied. We will focus on advanced-stage classification in the future when more MRI data can be obtained. In addition, we will take the impact of the tumor microenvironment into consideration while determining the depth of tumor infiltration. A more comprehensive system will be designed to fully utilize imaging resources and provide creative solutions.

There are some potential limitations of our study: (1) our experimental data were obtained from a single center and only sagittal T2WI images were used. Although the CAD achieved a good result, we believe that a model using a combination of various MRI images (T1WI, diffusion-weighted imaging (DWI), etc.) should be considered for future research. Additionally, we are open to collaborating with other centers to enhance the robustness and generalizability of our findings in future studies. (2) The ACC of using the object detection model to select the best MRI slices did not achieve satisfactory results, which we believe is mainly due to the insufficient amount of data used for training and thus the low generalization of the model. (3) The final CAD diagnosis results are strongly influenced by the performance of the semantic segmentation model since the UCLGA performs the MI depth calculation on the segmented image. The segmentation model based on Attention U-net could also be improved to get a higher ACC. At the same time, while our UCLGA solves most problems, the diversity of uterine shapes still leads to a small number of incorrect diagnoses. In this case, the experience of the doctor and the ability to think dialectically reflect the irreplaceability of human beings.

## Conclusions

This study implemented an end-to-end CAD system for early EC classification. The optimal MRI slices were selected automatically by an SSD-based detection model. The uterus and lesion area were localized and outlined by a multi-stage DL model method on MRI images. Finally, it accurately determined the MI depth by using an ellipse fitting algorithm to mimic the UCL. The results showed that the method has a diagnostic performance comparable to that of radiologists. This CAD method is more intuitive and interpretable than previous DL-based CAD methods.

## Materials and methods

### Patients and data preparation

The Institutional Review Board (IRB) of Fujian Maternity and Child Health Hospital in China (FMCHH) approved the retrospective study, and the requirement for informed consent was waived. 207 patients who underwent pelvic MRI examination in FMCHH during the period from January 1, 2018, to December 31, 2020, were included in this study after being pathologically diagnosed with early-stage EC. Patients were identified by using information from the hospital’s picture archiving and communication system (PACS). The exclusion criteria were as follows: (1) without a final pathologic diagnostic statement; (2) inability to pathologically confirm early-stage EC (FIGO stage IA or IB); (3) missing MRI data (no corresponding sagittal T2WI sequence). The total number of patients in the study was 154 (mean age 55.7 ± 9.7 years, 75 stage IA and 79 stage IB). All the included patients were confirmed by pathology as shown in Table 4.

**Fig. 7:**
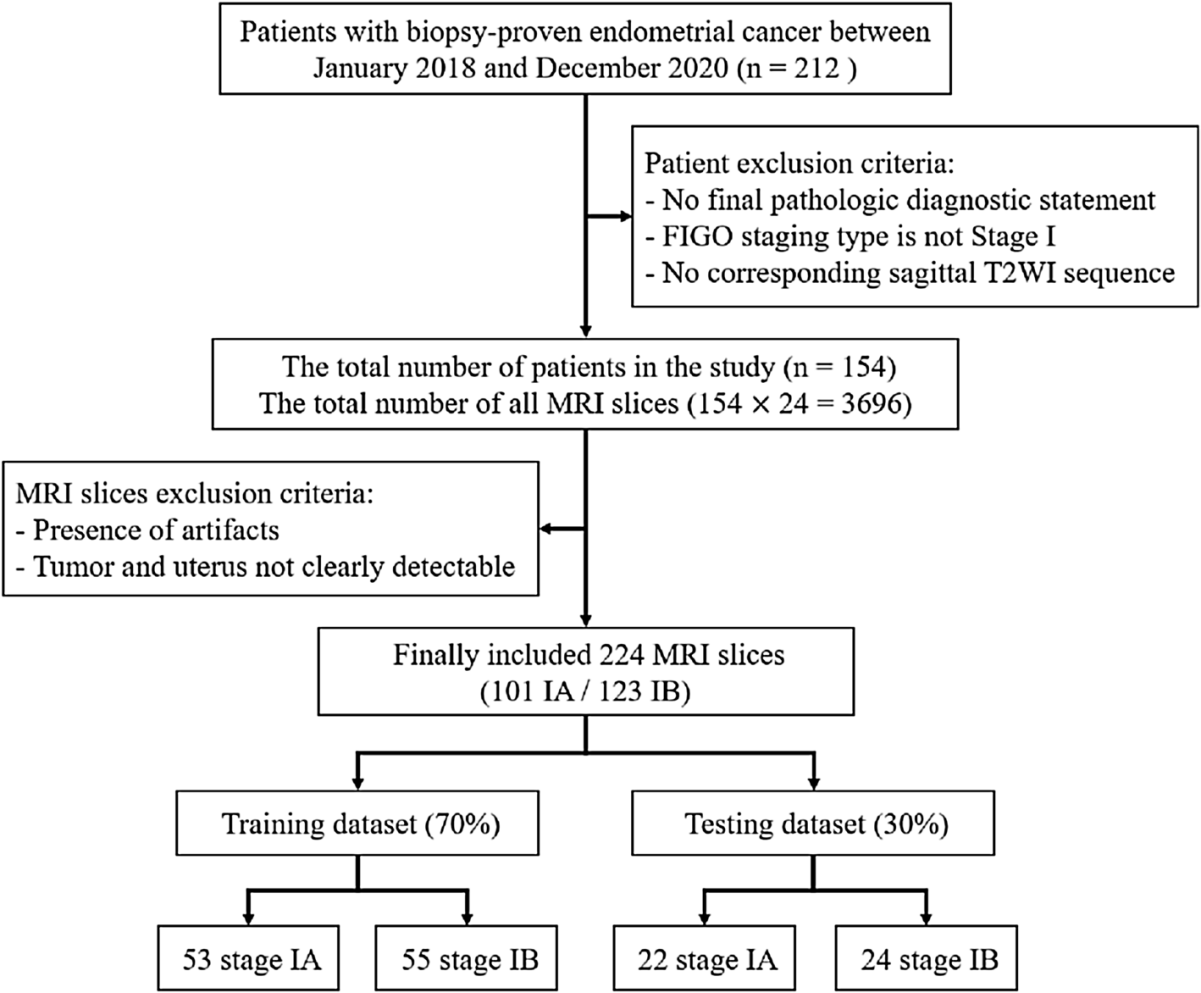
A flow diagram of the cohort selection

Subsequently, visual selection of MRI sequences (24 slices per sequence, for a total of 3696 MRI images) was performed by two experienced radiologists in a consensus manner and the following exclusion criteria were applied: (1) presence of artifacts; (2) uterus and tumor not clearly detectable on T2WI images. Radiologists usually select the MRI slice with the maximum tumor diameter as the central slice and 1–2 anterior and posterior slices of the central slice as the selected objects for analysis. Finally, the experimental data are 224 MRI slices (101 IA images, 123 IB images). The experimental data are randomly divided into the training dataset (70%) and the testing dataset (30%). The training dataset has 108 cases (53 stage IA/55 stage IB) including 156 images, and the test dataset has 46 cases (22 stage IA/24 stage IB) including 68 images. A flow diagram of the cohort selection is presented in Fig. 7.

The proposed methods are all based on a dataset composed of the aforementioned MRI images. Since this dataset is relatively small in terms of the number of images, data augmentation techniques, such as random horizontal flipping, random vertical flipping, and random scaling, were employed during the training process to enhance the model’s robustness and prevent overfitting [21].

**Table 4:** Clinical and pathological data summaries in training, and independent test group

Parameter	Training data (*n* = 108 )		Independent test data (*n* = 46)		
	Stage IA	Stage IB	Stage IA	Stage IB	*p* value
Subpopulation	53	55	22	24	
Age (year)	51.4 ± 8.9	58.9 ± 9.3	48.9 ± 10.2	58.5 ± 9.8	0.998
Pathological type					0.918
Grade 1	32	26	17	9	
Grade 2	19	23	5	12	
Grade 3	2	6	0	3	
Maximum diameter (cm)					0.913
<3	38	21	17	9	
$\ge$3	15	34	5	15	
Myometrial invasion					0.951
<50%	51	7	22	3	
$\ge$50%	2	48	0	21	
Mixed carcinoma$^{*}$					0.896
No	32	27	11	19	
Yes	21	28	11	5	

*Indicates the presence of other tumors, such as clear cell carcinoma, uterine fibroids, etc

### MRI protocol

All MRI examinations were performed using a 1.5-T MRI scanner (Optima MR360, GE Healthcare) with a phase-controlled oscillation coil. Before the examination, the patient’s bowel was defecated using a glycerine enema and had an appropriate urine holding (about one-half). To reduce bowel artifacts and motion artifacts caused by significant bowel movements, no enemas or slow-defecation medications were used for bowel movements. Eating was allowed (food cannot contain iron components) and no intramuscular injection of any medication was required. Ensure that the patient has no contraindications to MRI and no metallic foreign bodies on the body. It is especially important to ask whether the patient has ever had surgery or radiation chemotherapy. Whether the current status is menstrual or menopausal. The patient’s position was feet-first and supine in all cases. Keep the body in line with the bed of the MR scanner so that the scanning site is as close as possible to the main magnetic field and the center of the coil, the center of the coil to the pubic symphysis. Place a soft cushion on the lower abdomen to reduce motion artifacts caused by breathing. Simultaneously, ask the patient to raise both hands (ensuring they do not cross their hands to form a loop) and provide appropriate support using triangular cushions to ensure the patient completes the examination in a comfortable position. The fat-suppressed fast-spin-echo T2WI (FS FSE T2WI) sagittal sequence was selected for this study. The acquisition parameters of the MRI were as follows: repetition time/echo time [TR/TE], $5600-5700/65-70$ *msec*; bandwidth, 31.25*Hz*/*pixel*; thickness, 5 *mm*; flip angle, 160*degrees*; field of view, $280\,mm$; matrix size, $320 \times 224\,mm$; and image resolution, $512 \times 512 pixels$.

### MRI lesion labeling

**Fig. 8:**
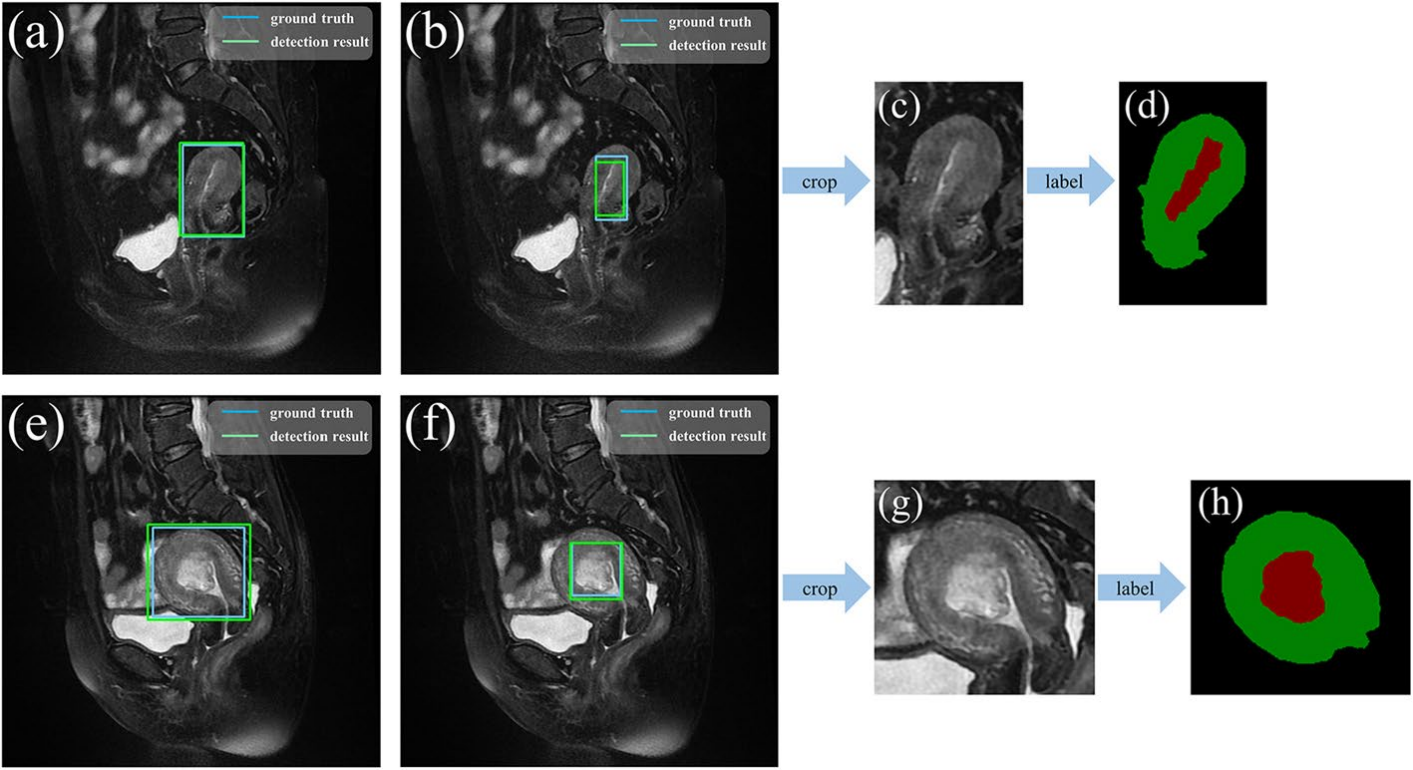
**a**, **b**, **e**, and **f** are the labeling and prediction of the object detection model. **a** and **e** Are uterus regions, **b** and **f** are tumor regions. **c** and **g** are cropped images based on the detection results. **d** and **h** are labeling of the semantic segmentation model (red is the tumor, green is the uterus)

Localization of ROIs in all MRI images and segmentation of ROI contours in cropped images in this study were performed by experienced radiologists (Chen’s team). For the object detection model, two rectangular boxes were drawn as labels for the dataset using labelImg (version 1.8.5), one including the uterus, and the other including the lesion structures (Fig. 8), and these borders were considered as the ground truth for the object detection model. For the semantic segmentation model, the edge contours of the lesion region and the uterine body were outlined using labelme (version 4.5.7), which was used as the label of the dataset, and these two contours were considered as the gold standard for the semantic segmentation model (Fig. 8).

### Proposed method

**Fig. 9:**
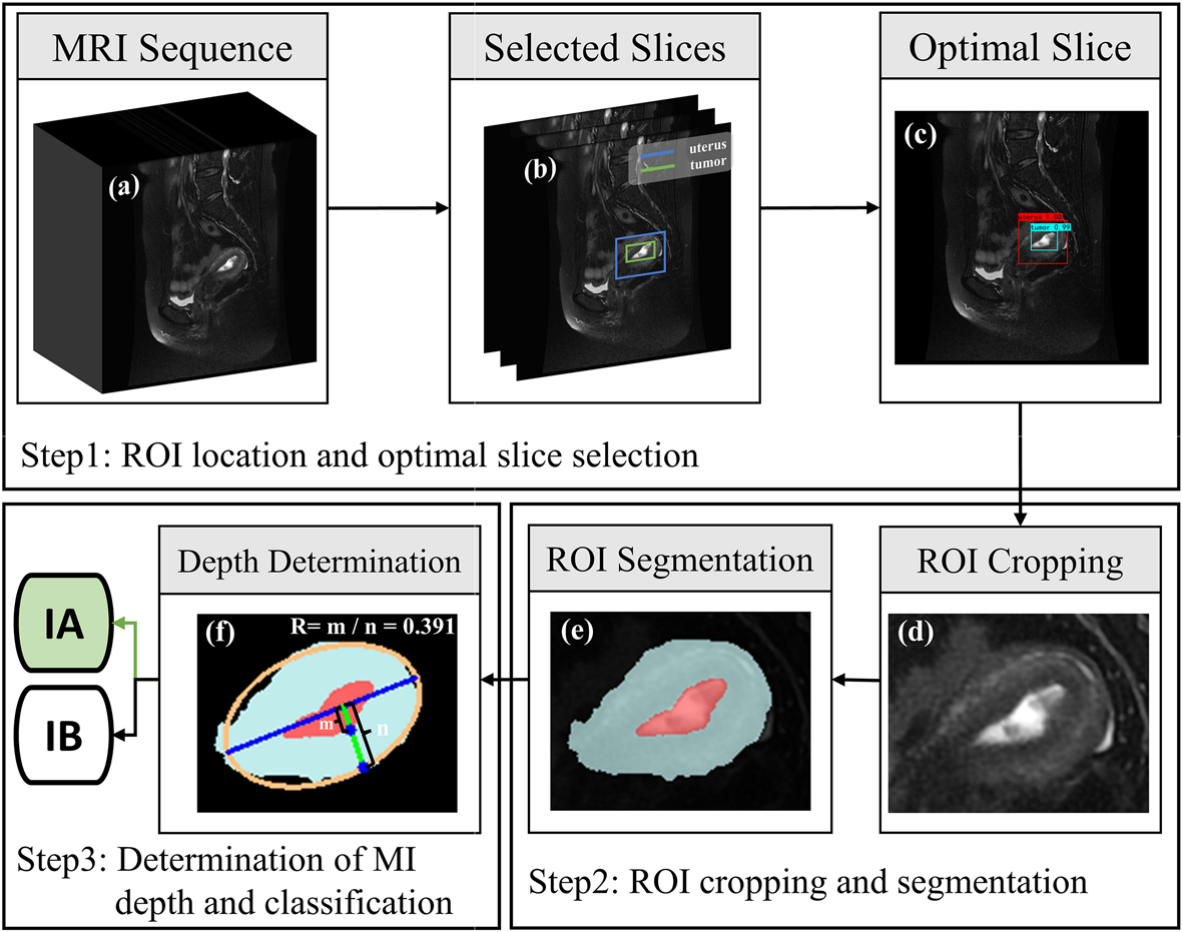
The flowchart of the proposed method. **a** is the original MRI image sequence. The object detection model based on the SSD algorithm is used to detect the ROI (uterus and tumor) (**b**). **c** is the optimal image that can clearly see the ROI. **d** is the cropped image, which only includes ROI. The semantic segmentation model based on Attention U-net is used to accurately predict the uterus (light blue region) and tumor (red region) of the cropped image **e**. **f** is the ellipse fitting algorithm that is used to generate UCL, and the R in (**i**) is the final prediction of the depth of MI

A DL-based multi-stage CAD method is proposed to evaluate the exact MI depth (Fig. 9). The object detection model based on the SSD algorithm is used to perform ROI detection on the original MRI image sequences (Fig. 9a). The MRI images that can clearly show the uterus and tumor are selected according to the confidence score of the detection results (Fig. 9b), and be cropped out according to the detection box (Fig. 9c). The cropped images (Fig. 9d) are fed into a semantic segmentation model based on the Attention U-net network for prediction (Fig. 9e). Then the ellipse fitting algorithm based on UCLGA is employed to generate the UCL on the segmentation map. The MI depth is obtained by the ratio of the tumor-UCL maximum length to the uterus-UCL maximum length. According to the criteria of the FIGO for determining the staging of early EC tumors, the depth of tumor infiltration less than 50% of myometrial thickness is identified as stage IA and greater than 50% of myometrial thickness is identified as stage IB [22]. Finally, the EC MRI image is classified as IA stage when MI is less than 0.5 and IB stage when MI is greater than 0.5.

**Fig. 10:**
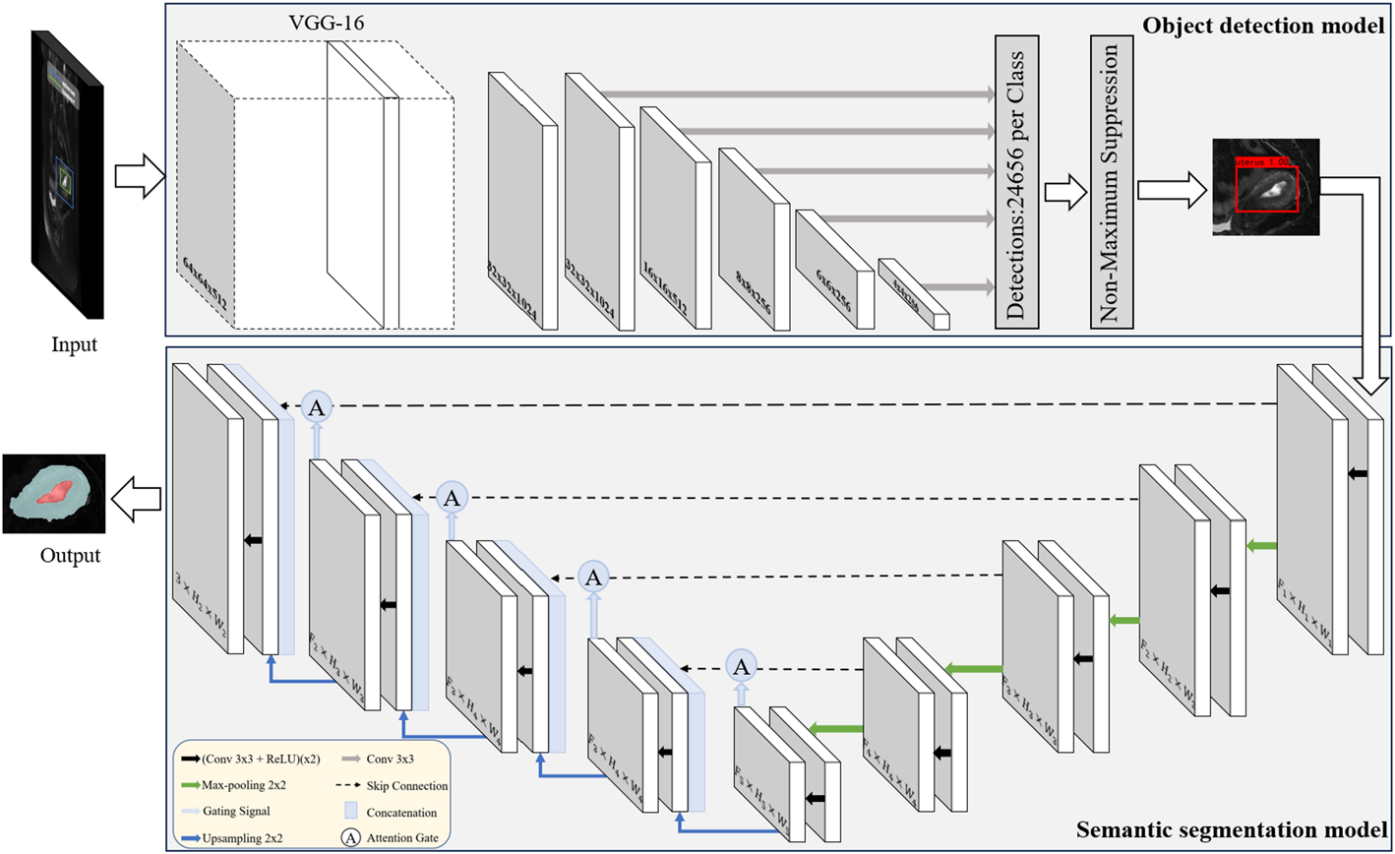
The architecture is used for object detection and semantic segmentation

#### Object detection model

The task of object detection is to locate instances of a certain class of semantic objects [23]. In this study, the SSD model [24] is employed to detect the bounding box of ROI in MRI images. The architecture of the model is shown in Fig. 10. SSD is a method for object detection in images using a single deep neural network. SSD extracts features from the image using VGGNet. Additional convolutional layers are then added on top of these features to generate feature maps at different scales. These feature maps contain information about objects of different sizes and scales, allowing SSD to detect objects of different sizes. Then it discretizes the bounding box output space at each location on the feature map, into a set of default boxes with different aspect ratios and scales. Each of these default boxes predicts the confidence level of its internal object class and the offset relative to the ground truth box. Finally, the proportion of positive and negative samples of the default boxes is controlled by non-maximal suppression and hard negative mining. Firstly, model parameters that were pre-trained on the VOC 2007 dataset were loaded. Secondly, the parameters of the first 21 layers of the pre-trained model for training were frozen in the first 50 epochs. Lastly, the parameters of the overall network were updated after 50 epochs of training, which achieved a higher training speed and better model performance. The original MRI images and bounding boxes outlined by radiologists were used as the input data to train the SSD model. The original MRI images were uniformly resized to 512$\times$512 and then fed into the object detection model for training.

#### Semantic segmentation model

Semantic segmentation is the ability to segment an unknown image into different parts and objects (e.g., beach, ocean, sun, dog, swimmer). Moreover, segmentation goes deeper than object recognition, because recognition is not necessary for segmentation [25]. In this study, the Attention U-net model [26] is used to segment the uterine and tumor regions of the input images. The Attention U-net is a variant of U-net that retains the original encoder–decoder structure as shown in Fig. 10. The encoding layer maps the input images to a latent representation or bottleneck, and the decoding layer maps this representation to the original images [27]. To concatenate the features of high and low levels together, skip-connection was added to the encoder–decoder network[21]. It is also boosted with attention gates to highlight better salient features passed through the skip connections [28]. First, the model parameters that were pre-trained on the VOC 2007 dataset were loaded. The parameters of the first 17 layers of the pre-trained model were frozen for training in the first 50 epochs, and then the parameters of the overall network were updated while training after 50 epochs, which achieved a faster training speed and improved model performance. The original MRI image is cropped according to the radiologist’s boxed-out uterine region and then fed into the semantic segmentation model for training. Due to the inconsistent size of the cropped images, a uniform size is required for semantic segmentation model training and prediction. To resize the image without distortion, this work supplements the image with gray bars of pixel value 128 around the image to unify the image to 256$\times$256, and the gray bars will be intercepted in the final prediction result.

#### Optimal slice selection

To solve the problem of requiring radiologists to manually select MRI slices that reflect the lesion, an object detection model is employed to automatically select MRI slices that can clearly see uterus and tumor from MRI sequence images. To begin with, the MRI sequence images of EC patients are fed into the object detection model for detection, and then three images of this sequence with the highest confidence scores of the uterus and tumor (predicted by object detection models) are selected as the screening results (Fig. 9a, b). The performance of the automated slice selection will be evaluated using the radiologist’s manually selected slices as positive labels. Since there are no quantitative criteria for radiologists to select the best slice, and usually more than one slice in an MRI sequence that clearly visualizes the uterus and tumor (One slice was selected by the radiologist as best slice in 24 patients, two slices were both selected by the radiologist as best slices in the other 22 patients). Three MRI slices were automatically selected by CAD for each patient. CAD1-accuracy is defined as the performance when CAD automatically selects only one slice to match the manually selected slices by the radiologist. Similarly, CAD2-accuracy is defined as the performance when CAD automatically selects two slices, and CAD3-accuracy is the metric used when any of the three slices suggested by CAD is among the manually selected slices by the radiologist. The implementation source code and experimental data of the module are available at https://github.com/mw1998/Optimal-MRI-selection.

#### UCL generation algorithm

Employing a single algorithm or model to determine MI depth is a great challenge due to the diversity of uterine shapes and tumor locations. Therefore, an algorithm for automatic UCL generation (Fig. 9) on the semantic segmentation map is proposed in order to calculate the MI depth. The UCL generation algorithm is described in Algorithm 1. To begin with, a line is obtained as the virtual UCL. Then, two maximum perpendicular lines to the UCL are determined. One line is the maximum thickness of the myometrium to the UCL and the other line is the maximum extent of tumor to the UCL. The ratio of the line lengths equals the depth of MI. A general formula of the ellipse is as shown in equation 1.

A. Fitzgibbon et al. proposed a direct least-squares fit an ellipse [29], which fits an ellipse specific to discrete data by minimizing the algebraic distance, subject to a constraint of $4ac-b^{2} =1$. It is easy to implement and extremely robust. Where *a*, *b*, *c*, *d*, *e*, *f* are the fitted ellipse parameters obtained from the set of points (x,y) extracted from the input uterine contour lines. The algorithm is applied to the uterine contour in the segmentation image and considered the fitted ellipse long axis as the UCL (Fig. 9f):

1 $$\begin{aligned} a{x^2} + bxy + c{y^2} + dx + ey + f = 0. \end{aligned}$$

Perpendicular lines are made at each point of the UCL, and the distance ratio of each perpendicular line to the intersection of the tumor border and the uterine border is calculated, and the maximum distance ratio is considered as the MI depth (m, n in Fig. 9f).


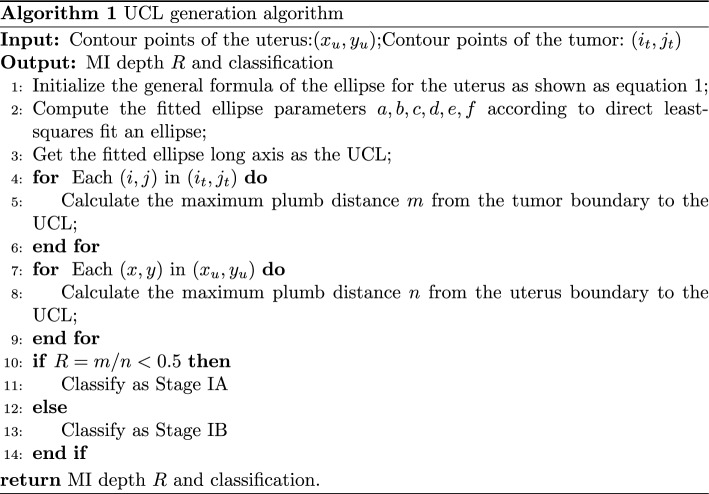


### Validation and statistics

A test dataset containing 68 images from 46 randomly selected patients is used to validate the performance of the CAD method. Given a patient with a sequence of sagittal T2WI images (the number of images varies from 19 to 23) is first fed into the object detection network to select the optimal MRI slices in which the tumor and uterus could be clearly visualized. Then, the radiologist-selected slices are cropped according to the detection boxes predicted by the object detection network and fed into the semantic segmentation network to obtain segmentation maps of the uterine region and the tumor region. Finally, UCL is generated using the UCLGA to yield the infiltration depth and classification of MI. Statistical analyses are performed on SPSS (version 26.0., SPSS Inc.) and p-values are obtained by *t*-test.

## Data Availability

The datasets generated during and analyzed during the current study are available at https://github.com/mw1998/Optimal-MRI-selection.
